# Joining forces for pathology diagnostics with AI assistance: The EMPAIA initiative

**DOI:** 10.1016/j.jpi.2024.100387

**Published:** 2024-05-31

**Authors:** Norman Zerbe, Lars Ole Schwen, Christian Geißler, Katja Wiesemann, Tom Bisson, Peter Boor, Rita Carvalho, Michael Franz, Christoph Jansen, Tim-Rasmus Kiehl, Björn Lindequist, Nora Charlotte Pohlan, Sarah Schmell, Klaus Strohmenger, Falk Zakrzewski, Markus Plass, Michael Takla, Tobias Küster, André Homeyer, Peter Hufnagl

**Affiliations:** a Charité-Universitätsmedizin Berlin, Corporate Member of Freie Universität Berlin and Humboldt-Universität zu Berlin, Institute of Pathology, Charitéplatz 1, 10117 Berlin, Germany; b Fraunhofer Institute for Digital Medicine MEVIS, Max-von-Laue-Straße 2, 28359 Bremen, Germany; c Technische Universität Berlin, DAI-Labor, Ernst-Reuter-Platz 7, 10587 Berlin, Germany; d QuIP GmbH, Reinhardtstraße 1, 10117 Berlin, Germany; e Institute of Pathology, University Hospital RWTH Aachen, Pauwelsstraße 30, 52074 Aachen, Germany; f Institute of Pathology, Carl Gustav Carus University Hospital Dresden (UKD), TU Dresden (TUD), Fetscherstraße 74, 01307 Dresden, Germany; g Medical University of Graz, Diagnostic and Research Center for Molecular BioMedicine, Diagnostic & Research Institute of Pathology, Neue Stiftingtalstrasse 6, 8010 Graz, Austria; h Vitasystems GmbH, Gottlieb-Daimler-Straße 8, 68165 Mannheim, Germany

**Keywords:** Digital pathology, Artificial intelligence, Standardization, Interoperability, Validation of algorithms, Explainability

## Abstract

Over the past decade, artificial intelligence (AI) methods in pathology have advanced substantially. However, integration into routine clinical practice has been slow due to numerous challenges, including technical and regulatory hurdles in translating research results into clinical diagnostic products and the lack of standardized interfaces.

The open and vendor-neutral EMPAIA initiative addresses these challenges. Here, we provide an overview of EMPAIA's achievements and lessons learned. EMPAIA integrates various stakeholders of the pathology AI ecosystem, i.e., pathologists, computer scientists, and industry. In close collaboration, we developed technical interoperability standards, recommendations for AI testing and product development, and explainability methods. We implemented the modular and open-source EMPAIA Platform and successfully integrated 14 AI-based image analysis apps from eight different vendors, demonstrating how different apps can use a single standardized interface. We prioritized requirements and evaluated the use of AI in real clinical settings with 14 different pathology laboratories in Europe and Asia. In addition to technical developments, we created a forum for all stakeholders to share information and experiences on digital pathology and AI. Commercial, clinical, and academic stakeholders can now adopt EMPAIA's common open-source interfaces, providing a unique opportunity for large-scale standardization and streamlining of processes.

Further efforts are needed to effectively and broadly establish AI assistance in routine laboratory use. To this end, a sustainable infrastructure, the non-profit association EMPAIA International, has been established to continue standardization and support broad implementation and advocacy for an AI-assisted digital pathology future.

## Introduction

Advances in precision medicine, enabled by improved diagnostic accuracy and novel therapeutics, have significantly improved clinical outcomes for patients. Pathology, a central diagnostic part of precision medicine, thus faces a substantial increase in workload.^1^, ^2^, ^3^ At the same time, there is a global shortage of pathologists.^4^, ^5^, ^6^AI-assisted image and data analysis could increase pathologists' productivity.^7^, ^8^, ^9^, ^10^ Researchers have published many promising algorithmic solutions.^11^^,^^12^ However, the path to wide clinical adoption is difficult. A core problem is a lack of standardization and interoperability for the seamless integration of image analysis methods into diverse image management and laboratory information systems. Commercialization and clinical implementation of pathology AI must overcome additional hurdles,^13^^,^^14^ namely the transformation of an idea into an AI prototype (which requires data acquisition), a validation process towards market readiness, and certification as a medical product. Finally, reimbursement and billing issues must be solved to generate revenue.

The EcosysteM for Pathology diagnostics with AI Assistance (EMPAIA) consortium was established in response to the 2019 innovation competition “Artificial Intelligence as a Driver for Economically Relevant Ecosystems” by the German Federal Ministry for Economic Affairs and Climate Action. EMPAIA's mission was to promote the ecosystem for AI in pathology by involving all relevant stakeholders and addressing issues of digitalization, standardization, legal and regulatory requirements, and billing. In addition, the sister project “EMPAIA Austria” was funded by the Austrian Research Promotion Agency to conduct related research on user interfaces and explainability.

The specific objectives of EMPAIA were:

1. Specify open interfaces for interoperable AI apps in pathology, taking into account requirements of different vendors.
2. Collaborate with diverse pathology labs (“reference centers”) to evaluate and obtain practical feedback on the EMPAIA developments.
3. Support AI vendors by open-source reference implementations of the interfaces and by recommendations on product development and regulatory affairs.
4. Develop explainable AI (XAI) approaches specifically for pathologists.
5. Transfer knowledge and facilitate exchange between all stakeholders.

**Fig. 1:**
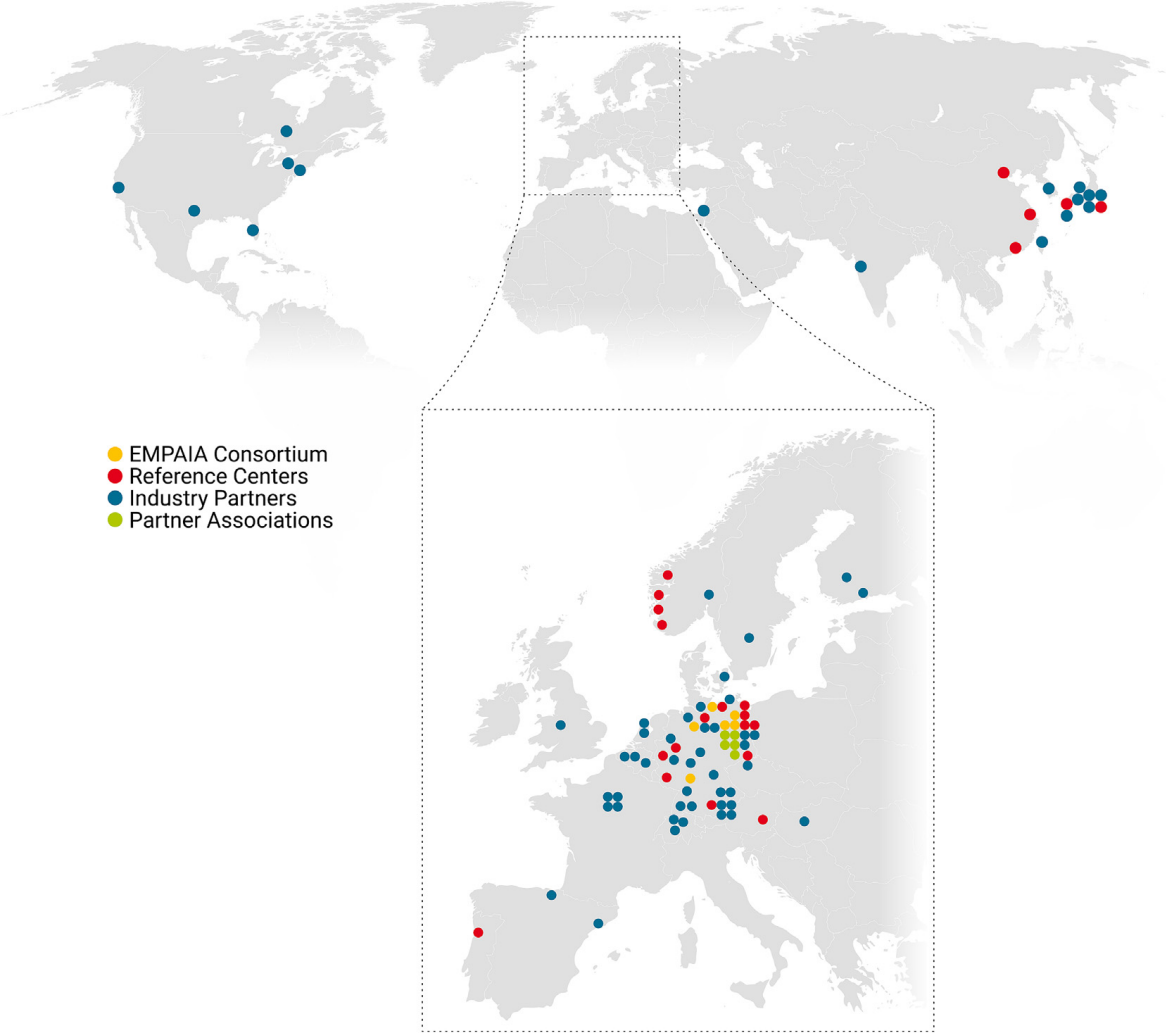
Locations of national and international EMPAIA partners. (World map adapted from Wikimedia Commons).^16^

The core EMPAIA team comprised 25 members, including pathologists, informaticians, mathematicians, technicians, designers, and supporting staff. Many international stakeholders have been involved from the beginning, including vendors of AI solutions/pathology software systems/scanners, diagnosticians from large and small clinical institutions, pharmaceutical companies, and AI researchers. Having started with 26 commercial partners and 5 partner associations, the network has grown substantially to 59 commercial partners, 5 associations, and 16 reference centers (Fig. 1). An updated list of consortium members, industry partners, and reference centers can be found on the project webpage.^15^

Here, we discuss the achievements of EMPAIA, including the corresponding lessons learned and their future continuation. We describe technical developments, such as the specification of unified interfaces for AI apps together with multiple industry partners and how the EMPAIA Platform can facilitate their adoption. In this context, we also describe how we collaborated with reference centers to test the developments in real workflow scenarios. We summarize our research findings for XAI in pathology to make analysis results more understandable and gain user acceptance. We describe how EMPAIA supported AI providers with regulatory issues, for instance, by publishing guidelines^17^ and initiating a service for the validation of AI solutions. We describe how we reached out to diverse audiences of the digital pathology ecosystem to increase visibility and engage relevant stakeholders. Lastly, we provide an overview of synergies with related initiatives and an outlook on how the newly founded non-profit EMPAIA International association will drive forward these activities in the future.

## Technical developments and evaluation

### Standardization

For efficient use in the diagnostic workflow, AI solutions must interact with various systems in the laboratory's IT infrastructure. The basis of this infrastructure is an AP-LIS, which manages case and sample information, including diagnostic results. An IMS handles slide scans for whole-slide images (WSIs), which can be implemented as a picture archiving and communication system (PACS) or vendor-neutral archive (VNA).^14^ In addition, there are pathology workstations with graphical user interfaces for WSI viewing and interacting with AI solutions, compute infrastructure for high-performance AI processing, AI application registries, and billing systems.

The use of AI solutions is severely hampered by a lack of interoperability.^18^ Some software systems already offer their own application programming interfaces (APIs) for executing AI solutions and exchanging input and output data. In this process, WSIs are usually represented according to proprietary file format specifications from various scanner vendors. AI developers must adapt their solutions to all these different APIs and WSI formats, leading to repetitive integration work and delays in the availability of AI applications, which drives up their costs. Many systems currently do not even offer any interfaces for AI solutions.^19^

#### The EMPAIA Platform

To improve this situation, we specified the EMPAIA App Interface, an open and vendor-neutral standard for integrating AI solutions into pathology software systems.^18^ The API was developed in close cooperation with associated industry partners, specifically considering their requirements. To facilitate the adoption of the EMPAIA App Interface, we published comprehensive documentation describing the technical implementation from the perspective of developers of AI apps and pathology software systems.^20^ In addition, we released the EMPAIA App Test Suite, an open-source toolbox for automatically checking the compliance of a given AI app.^21^ With the documentation and test suite, third-party developers were able to integrate and test their applications without further advice.

**Fig. 2:**
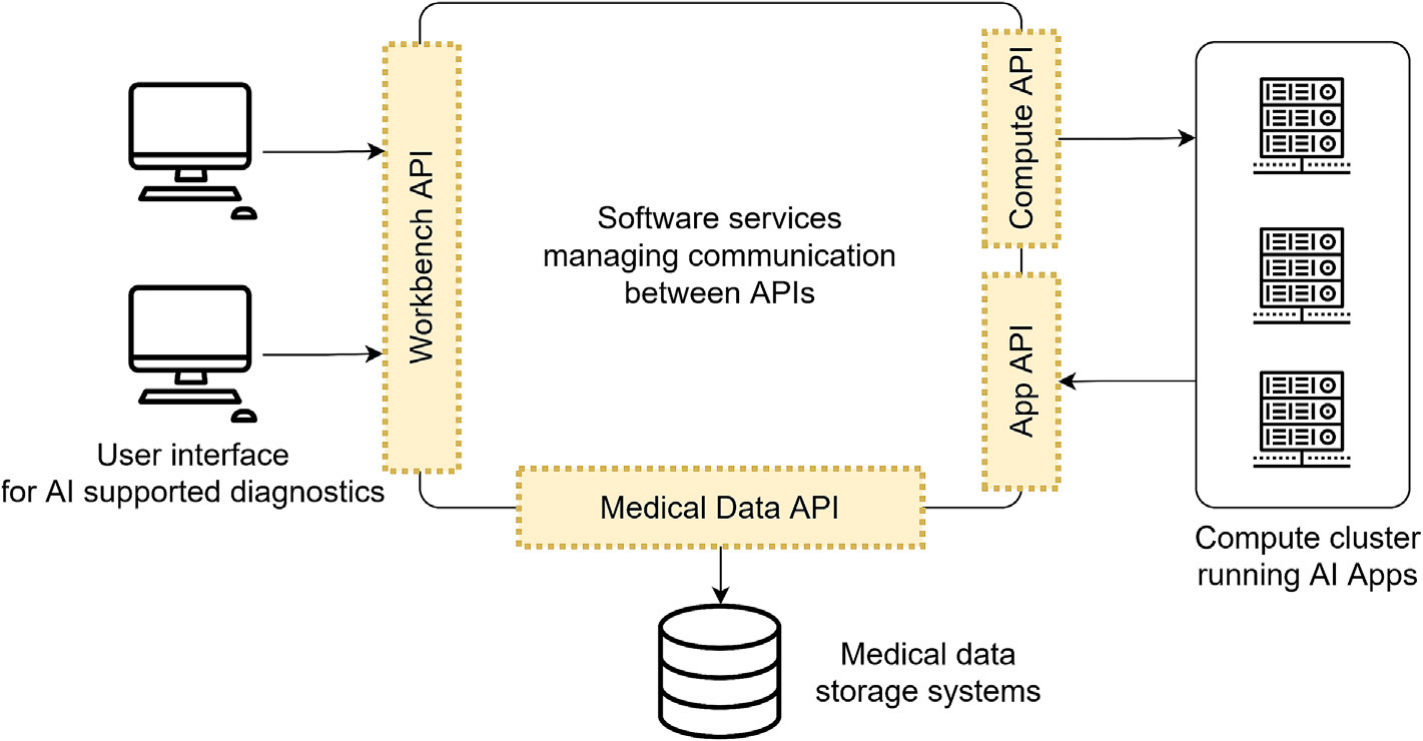
EMPAIA Platform architecture. Simplified diagram of the EMPAIA Platform architecture showing the main APIs serving as abstractions over clinical system software components.

The EMPAIA App Interface is embedded in the EMPAIA Platform (Fig. 2). Its highly modular architecture demonstrates how the different systems of the laboratory IT infrastructure can be integrated effectively to enable the use of AI.^22^ The platform provides ready-to-use implementations of critical components, such as a user workbench with a virtual microscope viewer and data management services. The platform is purely prototypical, neither certified as a medical product nor intended to compete with commercial pathology software systems. However, it is open source and subject to the industry-friendly MIT license,^23^ so both software system providers and laboratory IT administrators can use the platform as a blueprint and testbed for custom implementations.

The EMPAIA Platform comprises several APIs that represent abstractions of different software systems (Fig. 2). These abstractions allow for flexibility in the implementation of the technical components and enable combining different programming languages and database technologies while maintaining compatibility between the systems. The heart of each AI app is a model that takes image data and other parameters as input, processes the image and produces output data. EMPAIA specifies how such AI models are encapsulated using container technologies for uniform execution. AI models communicate via the EMPAIA App Interface to transmit input and output data. Equally important are user interfaces that allow pathologists to view WSI, interact with the AI solution, e.g., by drawing a region of interest (ROI), and render processing results. The EMPAIA App UI (user interface) concept allows AI vendors to bundle a custom web-based user interface with their apps, which can provide an optimized user experience for the specific diagnostic task. It also potentially reduces the effort required for regulatory compliance when integrating into new systems because AI models are usually validated in the context of the full diagnostic workflow. This diagnostic workflow includes the graphical user interface that displays AI results in a specific way and, depending on the AI app, might enable user interactions like drawing an ROI or selecting hotspots for further analysis. EMPAIA allows custom app user interfaces to be seamlessly embedded into browser-based pathology workstations and to connect with the underlying clinical system via the EMPAIA Workbench API.^20^

#### Lessons learned

Because the utility of the EMPAIA Platform is highly dependent on the acceptance and contributions of external AI companies, it was essential to pursue an agile development approach. First, a detailed requirements analysis was conducted together with AI companies to identify a small subset of features covering the minimum requirements of many products on the market. This allowed us to quickly release a minimum viable version of the platform. While this version was limited to the analysis of ROIs drawn by users in a WSI viewer, it enabled AI developers to create first usable prototypes of their apps and to provide early feedback for future development. Building on the minimum viable version, the platform was iteratively expanded and improved, for instance, by further annotation types, configuration options, or preprocessing functionality. The development process also revealed previously unknown requirements. In particular, the need to enable custom app user interfaces was only identified through feedback from regulatory experts. It made our original concept of a unified user interface for all apps obsolete.^22^

The inconsistent implementation of apps from different vendors proved to be particularly challenging. On the one hand, the apps were implemented based on different computing platforms and paradigms, e.g., as Windows-based client applications or Linux-based cloud applications. Only container technologies allowed all apps to be integrated uniformly into the EMPAIA Platform. On the other hand, the apps expected different data inputs: While some apps could open WSIs of different formats by themselves, others depended on corresponding functionality in the host platform. Therefore, the platform had to be highly modular to supplement missing functionality as needed. This requirement is fulfilled with a layered design of APIs that serve as abstractions of the underlying implementations. Furthermore, the reference implementation follows a microservice architecture that allows parts of the implementation to be replaced, updated or improved as necessary, without affecting unrelated parts of the platform.

#### Perspective

The EMPAIA Platform is continuously being extended to new requirements. To coordinate future developments, we set up the EMPAIA Community Group, an open communication forum for all stakeholders.^24^ Changes and extensions to the platform are discussed in a transparent and formal manner through EMPAIA Mod Proposals.^25^ One of the most important current proposals is adopting DICOMweb (see below). Another highly requested extension is the possibility of storing and visualizing pixel-wise data overlays on WSIs.^26^ Such extensions would improve communication with DICOM-based PACS systems and enable AI apps to provide explainability functionality, thus simplifying integration and increasing the adoption of AI in clinical practice.

Clinical systems (AP-LIS, IMS) currently used in practice still have to work with various proprietary WSI file formats because the DICOM WSI standard has yet to be widely adopted. The EMPAIA team designed a common API that serves as an abstraction of such file formats (including DICOM WSI files). This approach allowed for a quick start in development to deliver a practical solution but resulted in an API that is not specifically optimized for DICOM. Because DICOM is widely considered to be the future standard for WSI storage and transmission,^19^ the EMPAIA team is investigating a shift towards the DICOMweb standard on the API level as part of an upcoming platform release while still supporting the usage of proprietary formats in the backend.^27^

The IHE PaLM Technical Committee provides guidelines for Pathology and Laboratory Medicine (PaLM).^28^ For instance, the committee published the Digital Pathology Image Acquisition (DPIA) profile,^29^ describing the digitization and storage process for WSIs. The EMPAIA specifications complement the IHE proposals by specifying how AI apps can access image data after storage in a clinical system.

Adopting the open EMPAIA App Interface and building on the EMPAIA Platform is in the hands of the industry. As of December 2023, 14 AI apps from 8 companies have been integrated into the EMPAIA Platform,^30^ and more integrations are being actively developed. Four international vendors of pathology software systems are in the process of enabling the APIs in their products. A growing catalog of compatible AI apps has the potential to encourage more software system providers to undertake integration efforts to enable their customers to use such apps, which, in turn, will trigger network effects that will encourage more AI companies to join the initiative. The EMPAIA specifications impose strict requirements and a separation of concerns between clinical systems and AI apps. EMPAIA, therefore, offers the advantage of a unified approach for interoperability, reducing the multiplied efforts of integrating many heterogeneous AI apps of different companies with various clinical systems.

### Reference centers

For an ecosystem project, it was crucial to partner with a variety of laboratories that used apps via EMPAIA. These partnerships enabled a better understanding of the requirements for adopting and using AI in clinical routine, testing the platform in practical settings, identifying and understanding issues, and discussing the potential for improvement.

For this reason, EMPAIA collaborated with multiple national and international reference centers, including seven university hospitals, two hospital chains, and six larger private practice groups. The reference centers implemented and tested the EMPAIA platform onsite and provided feedback from a clinical/technical perspective. They also participated in a user study evaluating the image analysis apps available via the platform. In addition, the reference centers performed their own research in the context of EMPAIA.^31^, ^32^, ^33^, ^34^, ^35^ The centers were at very different stages of the digitization process, which created additional challenges but was crucial for accurately representing real-world conditions.

#### Platform deployment and usage

To evaluate the EMPAIA Platform, reference centers were provided either access to a deployment in the EMPAIA cloud or an on-premises offline deployment. The deployment included a prototypical data management application that is part of the EMPAIA Platform. This way, the evaluation could begin even before a full LIS and IMS integration was completed. The data management application performed client-side anonymization before data transfer, mitigating data protection concerns and avoiding unnecessary transfer of patient data.^36^ For the evaluation, the reference centers could run selected analyses on demand, i.e., by drawing ROIs to trigger computations. The example apps available comprised a mix of research use-only, non-approved, and CE-IVD-approved apps. For legal and licensing reasons, app usage had to be restricted to evaluation and clinical use was prohibited.

To familiarize pathologists and qualified staff with AI at the reference centers, we asked them to evaluate multiple apps integrated into the EMPAIA Platform. In this context, we collected structured feedback from these users on the perceived correctness of histopathological tissue analysis with AI-assisted assessment compared to human-only assessment and feedback about usability and bugs. Pathologists, medical students, and researchers, including users with extensive routine experience in pathology and digital natives, were included to cover a diverse range of users. Depending on the preferences of the reference centers, human-only assessment was performed using a routine microscope or pathology viewer software. This collection of user feedback focused on obtaining actionable feedback for iterative improvement and thus provides anecdotal results rather than unbiased quantitative performance data.

#### Lessons learned

Despite obvious advantages, such as reducing efforts in procuring hardware or deploying in-house infrastructure, using cloud services posed a significant problem for some institutions due to IT security vulnerability and privacy concerns. For cloud-based deployments, a privacy agreement had to be reached between reference centers and EMPAIA, ensuring the secure transfer of anonymized medical data. As some apps depend on external cloud services, such apps were unavailable in the on-premises deployments. This indicates a lose–lose situation known from the ongoing controversy over local vs. cloud solutions: vendors of cloud-only apps lose potential clients, whereas labs using only offline apps lose potentially valuable tools.

User feedback provided clues as to why satisfaction with app assistance still has room for improvement. Besides issues with image quality, which need to be avoided by optimized lab workflows, and outages of the EMPAIA infrastructure, feedback concerned issues concerning app results, app usability, and app usage as part of the current platform. After categorization, we clarified details with the reference centers, curated the feedback, and forwarded it, as appropriate, within EMPAIA or to the app developers for improvements.

One app yielded results that were obvious to be inadequate in several labs, and another yielded inadequate results in a single lab, possibly due to the incompatibility of WSIs generated with a specific scanner. One app was criticized for not yet computing diagnostically relevant quantities from the image analysis results, and one app was criticized for poor usability. On the other hand, one app was praised for its capability to detect and quantify weak signals barely visible to the human eye. These issues and feature requests were followed up with the vendors.

The process of drawing an ROI and waiting for the computation of analyses was often perceived as excessively slow, which occasionally compelled users to create ROIs that were too small for any meaningful analysis. This underlines the need for unobtrusively fast implementations and, e.g., running computationally expensive WSI-wide analyses before a pathologist starts interacting with the case. The latest EMPAIA API version supports such preprocessing for compatible apps. Manual correction of algorithmic results was viewed as cumbersome. App results that did not align with criteria present in pathological analyses comprehensible and assessable by pathologists (e.g., glandular units) were not trusted, underlining the necessity of suitable explainable AI approaches. This indicates the need for more communication and a collaborative app development process between developers and users. This should lead to a clearer alignment of user expectations, app capabilities, and presentation of algorithmic results. Moreover, users considered integration with LIS indispensable and this issue will need to be evaluated once available. Operating apps as isolated tools was not considered useful for routine diagnostics.

These findings provide valuable user feedback both for the improvement of individual apps and for the next steps of integration in an iterative manner.The evaluation so far has focused on single apps. Once combinations of apps are available to assess complete clinical cases with different stainings, usability studies will need to investigate to which extent user interaction should be homogenized across apps by different vendors. App performance should be evaluated in more detail, considering real patient cases with slides of different staining, i.e., requiring a targeted study setup for app validation. For this purpose, providing multiple apps via the same platform permits head-to-head comparisons and allows users to choose the optimal apps for their requirements.

## Explainability

Apps using AI algorithms are typically intransparent with respect to how results are obtained from the input images. There is an agreement on the need to control and understand AI solutions, especially when being used in potentially life-threatening domains such as medical diagnostics.^37^^,^^38^ However, there are multiple facets to explainability, e.g., which aspects should be explained to whom. Currently, no consensus exists on terminology and definitions of explainability of AI.

In EMPAIA, an AI application typically performs deep learning-based image analysis on WSIs. It, therefore, consists of one or more deep neural networks (models) and additional algorithms for pre-, intermediate- and post-processing tasks. While such deep learning models can achieve very high task performance (e.g., classification accuracy, intersection over union for segmentation tasks), model size and complexity are barriers to understanding their inner workings, making them a “back box.” Because their high-dimensional decision space and stochastic origin provide no inherent guarantee for correctness, one needs other means of estimating their function and reliability. Because the qualities of an explanation are highly dependent on the recipient of the explanation, the situation in which it is given and the goal it is expected to support, one needs to define this context in order to effectively discuss, select and measure an explainability method.^39^

We systematically investigated aspects of explainability within and around the context of using AI-based diagnostic support software in a clinical setting.^40^ This includes detailed knowledge of the stakeholders to provide customized explainability.^41^ We address different stakeholders, with a focus on operators of the AI software (pathologists), developers of AI software, decision-makers, and validators who need to make high-level decisions about the use of AI software. In the following paragraphs, we will lay out their use cases, goals, and our efforts towards providing explanations for each of these use cases.

The first and most prominent user group are the pathologists or technical assistants who are directly operating the AI application within tight time restrictions and with the goal of providing the correct medical diagnosis. In this group, explainability is a potential source of trust and safety. As pathologists are the ones who have to decide and are responsible for their decisions, they have an inherent need for information that helps them with their judgment.

We surveyed pathologists to learn about their general preferences and understandings regarding explainability methods and explanations.^40^ Besides favoring explanations that are close in style and content to what another pathologist would provide them with, we also noticed that some misinterpreted the explanations for segmentations. Besides the machine learning-related part of XAI, we also looked at human-centric evaluations. We found that user-friendly interfaces, enabling pathologists to explore causal relationships, play an essential role in keeping the human in the loop. However, such interfaces require more research and innovation.^41^

**Fig. 3:**
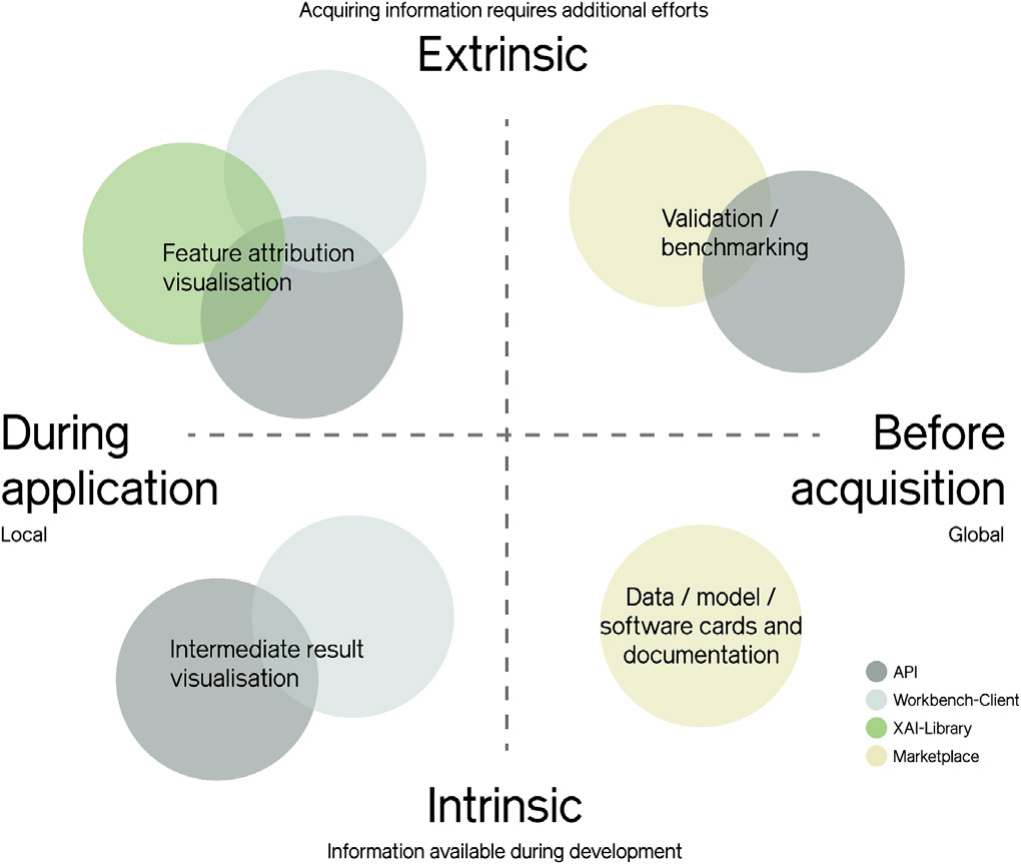
XAI approaches. Overview of different XAI approaches, the level they address and the project deliverables that have been affected or designed to support them. Local approaches provide explanations during usage of AI applications and directly address pathologists. Global approaches are supplements of AI applications that help decide whether they should be acquired in general.

Pathologists require explanations on a specific slide, sample, or case. These instance-based explanations are called local explanations. To provide XAI on the pathologists' desk, it has to be available and easy to use, e.g., by integrating it directly into the applications and user interfaces they use in their daily work, alongside the actual results of the ML algorithms. This can be achieved in many ways (Fig. 3), with varying degrees of explanatory value and understandability or the potential for misinterpretation if unfamiliar with the underlying method.

The second stakeholder and use case that we address are application developers. They aim to provide trustworthy, e.g., reliable, well-performing applications that follow legal and ethical regulations. Explainability methods can play a crucial role in reaching these goals. On the regulatory side, over the last few years, the EU published a set of directives and drafts for guidelines that already motivate and will create a strong demand for explainable AI. On the technical side, deep learning works stochastically and by example; if there were any unexpected and unwanted correlations (bias) in the training data, developers need ways to find out about such effects in order to address them.

Besides surveys, we investigated existing explainability methods applicable on image-based deep learning models. We developed a state-of-the-art model-agnostic explainability framework, including efficient optimization algorithms that speed up many existing model-agnostic methods by drastically reducing sampling efforts.^39^^,^^42^ We released these results together with implementations for several sampling-based XAI methods as an open-source library.^43^ Model-agnostic methods can be used to generate feature attribution heatmaps in cases without access to the model (external evaluation), or the model itself is not differentiable. Another reason can be the case of introducing new model architectures or transfer functions for which no reversible approaches exist yet. Model-agnostic methods come at a higher cost of execution. Still, they are cheaper to adapt to many different models, so they can help mass-validate vast ranges of different ML models when evaluating them before use in practical applications.

When several AI applications are run next to each other, consistent heatmap color gradients across apps should be used to avoid confusing users. To make the results of explainability methods, such as sensitivity maps, customizable, we created concepts and a software prototype for a standard of transmitting explainability information in a resolution and type similar to the original WSI. A crucial sub-concept in this API proposal^26^ was the distinction between content and its visual representation. As our previous observations highlighted, there is a need for customizable visualization depending on the shown information, stain color of the underlying WSI, etc. The second motivation are standardized user interfaces to enable the customizability mentioned above and consistency regarding color gradients. To realize this, our standard transmits only physically meaningful values together with semantic information required to select the appropriate color scheme to map to. Once implemented, these concepts must be verified in a usability study.

Finally, the third stakeholders we addressed are those who decide whether an AI application will be acquired or is safe to use in medical practice, e.g., hospital administrators, purchasing managers, and regulators. They typically require global explanations and information about how well an AI application performs on all of the patients rather than a single patient. This can also include information about the data that were being used for training the underlying models or the data that have been used to evaluate the models. Typically, this information is collected in model cards and can be shown in a portal like the prototype we developed in EMPAIA. A reliable and well-performed validation is the basis for supporting these stakeholders with such global explanations.

## Regulatory perspective

Apps in pathology used for diagnostic or therapeutic purposes are subject to the In Vitro Diagnostic Medical Device Regulation (IVDR)^44^ in the EU and the Code of Federal Regulations^45^ in the USA. Meeting these regulatory requirements is a major challenge when building AI products in pathology. Additional challenges include software quality, integration in laboratory IT infrastructure, business models, and reimbursement. To provide software vendors with advice on how to meet these challenges, we conducted extensive research and published an open guidance paper.^14^ In addition, EMPAIA is monitoring the pathology AI apps available on the market and their regulatory approval status in different jurisdictions and provides this directory online.^46^

To demonstrate the practical utility and obtain regulatory approval, AI apps must be validated appropriately.^47^ A major challenge in validating AI apps is compiling suitable test datasets. These must be sufficiently diverse to cover the extensive biological and technical variability of WSIs. They must also be sufficiently large to obtain statistically meaningful performance estimates. Moreover, test datasets must cover relevant subsets, be unbiased, and be sufficiently independent of datasets used for development.

In the past, there was little guidance available on how to compile test datasets, which was a major barrier to adopting AI solutions in laboratory practice. To change this, we organized the committee “Validation of AI Solutions,” consisting of >40 representatives from different stakeholder groups of the EMPAIA ecosystem, including pathologists, AI vendors, pathology software system vendors, and scanner vendors. The committee met in regular video conferences to discuss and define recommendations for creating and using test datasets based on literature research and experience. The results were published as an open guidance paper for AI developers and pathologists.^17^

To ensure that performance estimates are unbiased, AI apps must be tested on datasets that are independent of their training data and originate from different clinics and patients. Testing should ideally be conducted by an independent body with no conflicts of interest. For this reason, the EMPAIA App Validation Service was initiated in late 2022. AI vendors can submit their solutions to this service to have them tested against independent datasets curated by EMPAIA according to the proposed guidelines.^17^

In a first prototypical phase, the service provides validation of AI apps for PD-L1 immunohistochemistry (IHC) on WSIs in non-small cell lung cancer (NSCLC), detecting positive and negative tumor cells and calculating the Tumor Proportion Score (TPS). The dataset used for this purpose consists of over 200 slides from 22 cases that were stained with eight different antibodies. Reference TPS were provided by panels of three institutes. All slides were digitized using five different scanners, resulting in over 1000 WSIs. AI vendors do not receive the validation dataset itself, as sharing the dataset would no longer ensure an unbiased performance assessment for future AI solution submissions. Instead, AI vendors make their solutions compatible with the open EMPAIA Platform infrastructure,^22^ so that testing can be carried out autonomously by the EMPAIA App Validation Service. As a result, vendors are provided with a report of the specific strengths and shortcomings of their apps with regard to different tissue regions, stains, and scanner types.

## Communication and knowledge transfer

### Public relations

For an ecosystem project, it is crucial to be visible to find additional partners, in our case, app developers, LIS developers, and laboratories, and to attract the attention of future end-users, i.e., pathologists and decision-makers. These primary target groups are a diverse audience with backgrounds in computer science, management, and medicine. Five main channels were used to disseminate information: website, newsletter, social media, peer-reviewed publications, and conferences. Project news included events, publications, API updates, software, documentation releases, and the availability of EMPAIA Academy material.

Besides communication to the general public, we also disseminated project results to the scientific community in articles providing a project overview,^13^ describing aspects implemented in the EMPAIA platform,^18^^,^^36^^,^^48^ giving recommendations to different stakeholders,^14^^,^^17^^,^^22^^,^^49^ and presenting other EMPAIA-related research.^39^, ^40^, ^41^, ^42^^,^^50^, ^51^, ^52^, ^53^ Furthermore, congresses and events worldwide presented opportunities to showcase EMPAIA. These events allowed talking to media representatives, pathologists, and AI experts and to gain visibility and exchange information about the project and current developments in the field of digital pathology to establish and intensify networks.

### EMPAIA Academy

Activities involving stakeholders to deepen their shared knowledge of digital pathology were deemed crucial because the initiative intends to link people and software systems. We have grouped these programs together under the name “EMPAIA Academy.” We provided medical experts with an understanding of the basics of data science and AI development. Conversely, we showed AI developers the challenges of quality diagnostics and the legal considerations involved in creating advanced software and interfaces for a medical field. The EMPAIA Academy offered two types of workshops: lectures by selected invited domain experts and hands-on sessions at conferences, which provided insight into the technical activities behind data preparation and training of machine learning models.

Two rounds of half-day courses, IT/AI for pathologists and pathology for IT/AI specialists, were delivered as online webinars with free registration and recordings made available online to facilitate attendance. Basic courses covered laboratory workflow, pathologists' diagnostic work, introduction to WSI scanning, examples of AI applications in pathology, and best practices for algorithm development (for IT/AI specialists), as well as an introduction to machine learning, application of such techniques in pathology, and examples of AI use in different subspecialties (for pathologists). Advanced courses covered the productization of AI algorithms, an overview of existing products, and the regulatory and legal challenges of using AI in pathology. The hands-on sessions were held as pre-conference workshops at the European Congress on Digital Pathology in 2022 and 2023 and material was made available online afterwards.^54^, ^55^, ^56^ They consisted of several parallel tutorials, each covering different facets of computational pathology. Designed as activities requiring little prior knowledge of programming or pathology, the tutorials included interactive analysis of WSIs, automated image processing techniques, approaches for patch-based image analysis, and AI-based image classification. During the workshops, collaboration and discussions in small groups led to a lively atmosphere and networking. These workshops also stood out from the industry pre-conference workshops in that they provided knowledge to participants with no commercial interest.

## Discussion

Expectations for AI support in diagnostic pathology are high but are currently not being met. This is due to technical and regulatory challenges in translating research results into clinical diagnostic products and the lack of standardized interfaces between systems, among other reasons. We aimed to overcome these hurdles by developing standard interfaces for integrating AI apps into the workflow and by using these apps in routine clinical diagnostics, by supporting the validation and certification of AI apps, by fostering the exchange of knowledge between stakeholders (pathologists, algorithm developers, vendors, scanner vendors, and AI app vendors), and by improving the explainability of the results.

Starting from a digital pathology landscape with only a few proprietary interfaces to connect individual pathology workstations or IMS with AI apps from selected vendors, EMPAIA created the first open and vendor-independent interface specification. This was subsequently adopted by multiple vendors and tested in reference centers with encouraging results, providing a proof of concept of how different AI solutions can be integrated in a standardized way. The EMPAIA interface specification is being continuously extended to better integrate AI solutions into the laboratory workflow, e.g., by further improving DICOM interoperability and supporting different licensing models for billing (e.g., cloud-based and on-premises, time-based, and usage-based).

By collaborating with reference centers, a number of pathologists were able to familiarize themselves and gain experience with AI. In addition, many pathologists benefited from the knowledge transfer at the EMPAIA Academy. Similar to previous introductions of new methods in pathology (e.g., IHC, fluorescence in situ hybridization, molecular diagnostics), it will be important to expand training programs for clinical users in cooperation with pathologists' committees and organizations.

We have emphasized our position as an open and vendor-independent ecosystem by making the educational materials freely available and releasing the software developed for the EMPAIA Platform under the industry-friendly MIT open-source license. As a side-effect, this also allows the reuse of components and the adoption of interfaces beyond the intended purpose of making commercial AI apps available for routine pathology. It is conceivable that our developments could be used to create a platform for automated comparison of algorithms, to enable side-by-side comparison of apps in terms of quality of results, computational performance, and user experience, and to provide access to research apps, e.g., for clinical studies.

Obtaining FDA or IVDR approval for AI apps in DP is a major hurdle, especially for small vendors. This is sometimes circumvented by marketing apps for research use only, making it even more important to support the steps necessary for clinical use. While the first AI applications have been certified by the FDA or according to the IVDR in recent years, it is still not very clear what specific requirements vendors must meet. Therefore, we aimed to remove uncertainties about regulatory processes and, in particular, support the compilation of test datasets by deriving recommendations from the information available so far. We expect that the standardization of interfaces, as provided by EMPAIA, will simplify and accelerate certification. In addition to recommendations, we have created a first validation dataset (>1000 WSIs from >200 slides) as the basis for a service where AI vendors can obtain independent, external validation of their image analysis apps.

### Lessons learned

The EMPAIA initiative attracted a great deal of interest from the industry, as all market participants were facing more or less the same problems. We succeeded in opening up a pre-competitive space for interface development. This concept has already been successfully applied many times, e.g., with DICOM in the medical domain or the USB interface in consumer electronics. In this process, we underestimated the persistence with which proprietary approaches are pursued to achieve market advantages. It took considerable effort, repeatedly emphasizing that “we are not selling anything,” and demonstrating cooperation with various vendors before EMPAIA was no longer perceived as a potential competitor.

Setting up the reference centers also proved to be a significant administrative burden. Hardware acquisition, whether for a research project or not, must be processed according to the applicable European, national, and local tendering rules. This, combined with hardware availability bottlenecks due to the SARS-CoV-2 pandemic and the efforts of integration in local IT infrastructure, led to very long delays in establishing the reference centers. In addition, complex data protection agreements were required. Although no identifying patient data (including case numbers) was exposed to the image analysis apps, cloud use was not acceptable to all reference centers. Conversely, not all vendors provided on-premises implementations of their solutions. These difficulties are characteristic of the introduction of digital pathology in connection with AI apps by commercial providers and often result in stalled or abandoned deployments.

Once installations were established in the reference centers, getting thorough user feedback proved to be an additional challenge. Diagnosticians are under a lot of time pressure and are only able and willing to participate in evaluations, e.g., if the effort is reasonable. Even when communicated clearly, the fact that the platform, the apps provided, and the workflow integration are still under development can lead to a negative overall impression of AI and image analysis support. Combined with delays in implementation and improvements, this may also reduce enthusiasm for participating in further evaluations.

### Related initiatives

EMPAIA is one of several large-scale international initiatives that aim to support the development and use of AI solutions in pathology or other medical domains (Table 1). The initiatives complement each other and can build on each other's work in different ways.

**Table 1:** Large-scale initiatives that aim to support the development and use of AI solutions in pathology or other medical domains (alphabetical order; EU: European Union, UK: United Kingdom; DE: Germany, USA: United States of America; PF: public funding; IF: industry funding; NPO: nonprofit organization).

Name	Timeframe	Country/ Region	No. of partners	Type	Scope
BIGPICTURE^62^	2021–ongoing	EU	>20	IF, PF	Repository
EMPAIA / EMPAIA International e.V.^15^	2019–2023/ongoing	DE, global	>20	IF, PF, NPO	Standardization, interoperability, education, AI, regulatory, validation
European Cancer Imaging Initiative (EUCAIM)^63^	2023–ongoing	EU	>20	PF	Hub, repository, federated analysis (focused on radiology and genomics)
ITU Focus Group on Artificial Intelligence for Health (FG-AI4H)^60^	2018–ongoing	Global	n/a	PF	Standardization, regulatory
iCAIRD^66^	2018–2023	UK	5–20	PF, IF	Hub, AI development for radiology and pathology
IHE AI Interest Group for Imaging (AIGI) Task Force^59^	Ongoing	EU	n/a	IF	Interoperability, standardization
IHE PaLM^58^	2016–ongoing	EU	n/a	IF, NPO	Interoperability
Imaging Data Commons (IDC)^67^	Ongoing	USA	5–20	PF	Repository
ISO (ISO/AWI 24051–2)^61^	Ongoing	global	n/a	NPO	Regulatory, standards
NEMA Medical Imaging Technology Association (MITA)^57^	Ongoing	USA, global	>20	IF	Regulatory, standards, advocacy
PathLAKE^65^	2019–ongoing	UK	>20	PF, IF	Hub, repository, digitalization
PIcc (Pathology Innovation Collaborative Community - Alliance for Digital Pathology)^68^	2019–ongoing	USA, global	>20	IF, PF	Regulatory, standards

Several initiatives are dedicated to standardization and the creation of interoperability. The long-established NEMA Medical Imaging Technology Association (MITA)^57^ and IHE PaLM^58^ initiatives are working on standards for the representation and exchange of digital pathology images and structured metadata. The best-known example is the DICOM standard coordinated by NEMA MITA. The EMPAIA interfaces are already aligned with these standards and compliance is being further extended to improve the interoperability of input and output data from AI solutions (see section “Technical Developments and Evaluation”). Like EMPAIA, the IHE AI Interest Group for Imaging (AIGI) Task Force^59^ is also developing standards for using and integrating AI applications into end-user systems, but for radiology rather than pathology. As this task force started later, it can learn from the experiences of the EMPAIA project and endeavor to achieve interoperability between AI solutions in radiology and pathology from the outset. The joint support of the DICOM standard will facilitate this process.

The ITU Focus Group on Artificial Intelligence for Health^60^ (FG-AI4H) is working to create a standardized assessment framework for AI solutions in medicine, including pathology. The EMPAIA interfaces can prove useful here, as they make testing AI solutions from different manufacturers much easier within a standardized evaluation framework. At the same time, the EMPAIA recommendations for the creation and use of test datasets can help to obtain meaningful evaluation results.^17^ The ISO Technical Committee 212 is working on the ISO/AWI 24051–2 guidance document^61^ on the digitalization and processing of digital WSIs and their analysis using AI. The open guidance papers published by EMPAIA^14^^,^^49^ can provide a basis for this.

Lastly, multiple large consortium initiatives focus on building centralized or federated data repositories for pathology images (e.g., BIGPICTURE,^62^ EUCAIM,^63^ NCI Imaging Data Commons,^64^ PathLake^65^). By providing access via the standardized EMPAIA interfaces, these initiatives can enable the direct application of various AI solutions to their datasets, e.g., to automatically derive tissue parameters or to evaluate the AI solution on external data.

### Outlook

Despite the technical advances and guidelines developed over the past years, three major hurdles remain for the widespread adoption of AI in routine pathology. Obtaining regulatory approval remains a resource-consuming process for AI vendors. This will also be important to encourage updating products with the newest technologies, which is particularly relevant in the AI field. The process can be simplified through better guidance but will continue to require some effort. Digitization of pathology laboratories is a prerequisite for applying AI and remains a necessary major upfront investment in equipment and workflow adaptation by the lab. Enhancing existing guidelines with more experience and evidence can help to simplify the implementation to some extent. Reimbursement for using digital or AI-assisted pathology to cover additional costs, which could exceed what can be saved by more efficient workflows, will typically need to be decided nationally. Evidence of value and lobbying work can help make the use of AI financially viable.

Several standardization and regulatory issues require further development and sustainable engagement beyond the end of the publicly funded project. It is for this purpose that we have established the non-profit EMPAIA International Association. Open to all stakeholders in the digital pathology ecosystem, this association will remain vendor-independent. Its funding will come from membership fees, participation in publicly funded research projects, and the provision of services to its members, such as scanner benchmarking, provision of validation data, and interface implementation support. The association will focus on solving problems that require the cooperation of different stakeholders and neutrality towards all stakeholders, a prime example being the successful implementation of open interfaces for pathology-AI solutions. Therefore, a key goal of the association will be to further develop the EMPAIA Interfaces to meet new or expanded requirements of AI solutions and pathology software systems.

The high number of participants, i.e., pathologists, industry, researchers, legal and quality assurance associations, etc., in the EMPAIA Academy events, has demonstrated the demand for continuing education on AI in pathology. The Academy format, open to all interested parties, will be continued. In addition, the association will offer open online training programs for using and developing AI solutions with contributions from leading AI researchers and companies.

EMPAIA International works closely with a growing number of international reference centers and the stakeholder ecosystem to advance the use of AI and ensure the practicality of future developments, in particular by supporting the commercialization of AI prototypes and the validation of AI solutions. This way, the association will facilitate the development, marketing, and establishment of AI solutions in pathology.

## Contributions

Conceptualization: NZ, LOS, TRK, AH, PH; Methodology: NZ, LOS, TK, KW, TB, PB, RC, MF, CJ, TRK, BL, NCP, KS, FZ, MP, CG, AH, PH; Software: TB, CJ,MF, BL, KS, TK; Investigation: LOS, SS, FZ; Writing - Original Draft: NZ, LOS, TK, KW, TB, RC, MF, CJ, TRK, BL, NCP, KS, MP, CG, AH, PH; Writing - Review & Editing: NZ, LOS, TK, KW, TB, PB, RC, MF, CJ, TRK, BL, NCP, SS, KS, FZ, MP, MT, CG, AH, PH; Visualization: LOS, CJ, MP, CG; Project administration: PH, NZ, AH, CG, KW, MT; Funding acquisition: PH, NZ, TRK, AH, CG, KW. All authors read and approved the final version of the paper.

## Funding

This work was supported by the German Federal Ministry for Economic Affairs and Climate Action via the EMPAIA project (grant numbers 01MK20002A, 01MK20002B, 01MK20002C, 01MK20002E, 01MK20002F) and from the 10.13039/501100004955Austrian Research Promotion Agency under grant agreement No. 879881 (EMPAIA). CG has received additional funding via the European Union's Horizon 2020 research and innovation programme under grant agreement No. 101079183 (BioMedAI). TB has received additional funding from the German Federal Ministry of Education and Research in the projects PROSurvival (01KD2213D) and Inter-Agent (16DHBKI048). PB has received additional funding from the 10.13039/501100000781European Research Council (Consolidator Grant No 101001791), the German Federal Ministry of Education and Research (STOP-FSGS-01GM2202C) and the Innovation Fund of the Federal Joint Committee (Transplant.KI, No. 01VSF21048). MP has received funding via the European Union's Horizon 2020 research and innovation programme under grant agreement No. 101079183 (BioMedAI). TK has received additional funding from the German Federal Ministry of Labour and Social Affairs (Go-KI project DKI.00.00032.21).

## Declaration of competing interest

The authors declare the following financial interests/personal relationships which may be considered as potential competing interests:

Sarah Schmell and Falk Zakrzewski are employed by an AI vendor of which one app was included in the evaluation. The other authors declare that they have no known competing financial interests or personal relationships that could have appeared to influence the work reported in this article.
